# Multifractal characteristics of soil particle size distribution of abandoned homestead reclamation under different forest management modes

**DOI:** 10.1038/s41598-024-59466-w

**Published:** 2024-04-17

**Authors:** Tingting Meng, Jichang Han, Yang Zhang, Yingying Sun, Zhe Liu, Ruiqing Zhang

**Affiliations:** 1 Shaanxi Provincial Land Engineering Construction Group Co., Ltd, Xi’an, China; 2 Institute of Land Engineering and Technology, Shaanxi Provincial Land Engineering Construction Group Co., Ltd., Xi’an, China; 3 Key Laboratory of Degraded and Unused Land Consolidation Engineering, Ministry of Natural Resources, Xi’an, China; 4 Shaanxi Engineering Research Center of Land Consolidation, Xi’an, China; 5 Land Engineering Technology Innovation Center, Ministry of Natural Resources, Xi’an, China; 6 Key Laboratory of Cultivated Land Quality Monitoring and Conservation, Ministry of Agriculture and Rural Affairs, Xi’an, China

**Keywords:** Homestead reclamation, Forest management mode, Soil particle composition, Soil particle-size distribution, Single fractal, Multifractal dimension, Solid Earth sciences, Geophysics

## Abstract

In this study, fast-growing poplar reclaimed from abandoned homestead in Xixian New District, Xi’an City, Shaanxi Province, was used as the research object to explore the multi-fractal characteristics of soil particle size distribution under different management modes of abandoned land (control), irrigation, fertilizer irrigation and mixed fertilizer irrigation. The results showed that the mean values of soil clay, silt and sand in abandoned land were 14.58%, 81.21% and 4.22% respectively, 14.08%, 79.92% and 5.99% under irrigation, 15.17%, 81.19% and 3.64% under fertilizer irrigation, and 16.75%, 80.20% and 3.05% in mixed fertilizer treatment. From 40 cm, with increasing soil depth, soil clay particles increase under irrigation, fertilizer irrigation, and mixed fertilizer irrigation modes. The single fractal dimension of soil particle size distribution (*D*) in each treatment ranges from 2.721 to 2.808. At 60–100 cm, *D* shows fertilizer irrigation > mixed fertilizer irrigation > irrigation > abandoned land, indicating that fertilization and irrigation can increase the fine-grained matter of deep soil particles and reduce soil roughness. Compared with abandoned land, under irrigation, fertilizer irrigation and mixed fertilizer modes the capacity dimension (*D*_*0*_), entropy dimension (*D*_*1*_), correlation dimension(*D*_*2*_), shape characteristics of the multifractal spectrum (*Δf*) and overall inhomogeneity of the soil particle size distribution (*D*_*0*_*–D*_*10*_) indicate an uneven distribution of soil particle size; fractal structure characteristics of soil (*D*_*−10*_*–D*_*0*_) indicate a simplified soil structure, and degree of dispersion of soil particle size distribution (*D*_*1*_*/D*_*0*_) indicates that soil particle size is distributed in dense areas. Pearson correlation analysis showed that D was significantly correlated with clay, sand, *D*_*0*_*–D*_*10*_, soil organic matter (SOM) and soil available phosphorus (SAP) (*P* < 0.05). Stepwise regression analysis showed that clay was the main controlling factor of *D* and *D*_*0*_*–D*_*10*_ changes. The research results can provide some potential indicators for the quality evaluation of abandoned homestead reclamation.

## Introduction

Land serves as the foundation for human survival and development, supporting both human production and human life. However, with limited arable land resources, China has long suffered from insufficient land area and lack of reserve resources. Therefore, it is crucial to improve the quality and efficiency of land resource utilization^1,2^. Given the global trend of land reuse and the need to address the conflict between land supply and demand, land reclamation has emerged as the most effective solution to alleviate the tension between people and land. In recent years, urbanization and rural development have led to a growing problem of abandoned and unused rural residential land, resulting in a significant waste of land resources^3–5^. This has attracted the attention of policymakers and scientists in China, as the reclamation of abandoned homesteads offers a practical and viable approach to supplementing arable land reserves.

Land reclamation began in Europe and the United States with the development of industry, while the rise time of reclamation-related work and research in China lags behind that of foreign countries. Since the regulations on land reclamation promulgated by the State Council of China in 2011, research on land reclamation in China has gradually increased. Previous research reports on land reclamation mainly focus on coastal reclamation, such as the USA^6^, the Netherlands^7^, Japan^8^ (Suzuki.,2003), China^9–11^and Mexico^12^, which have carried out coastal reclamation for agriculture, mariculture, industrial use, urban development, and recreation. In addition, reclamation of mining areas is also a research hotspot, such as Li et al.^13^ and Gao et al.^14^ studied the methods and effects of reclamation in mining areas. However, there are few research reports on the reclamation of abandoned homesteads at home and abroad.

In recent years, the rate of homestead reclamation has increased year on year. However, because the homestead soil has been covered and embedded by the dump body for a long time, there is no accumulation of organic matter throughout the year, which leads to the destruction of soil structure, poor nutrient status, even the loss of some functions and properties, and low production capacity. It is urgent to rapidly improve soil fertility in this kind of reclaimed land through biological measures and fertilizer cultivation measures^15–17^. Biological measures mainly refer to the cultivation of herbaceous or woody plants, which not only provide ecosystem services such as microclimate improvement, soil improvement and reduction of wind speed and evapotranspiration, but also increase farmers’ income. These measures have become a popular method of land restoration for farmers^18,19^. Fast-growing poplar is a clone of Populusnigra L, which grows rapidly, has strong ability to resist natural disasters, and can quickly improve site conditions^20,21^. It is widely used in land restoration^22,23^. The input of organic matter as an effective method to increase soil organic matter, Bednik et al.^24^ showed that biochar can help to improve the fertility of sandy substrates and provide suitable conditions for vegetation restoration during reclamation. Li et al.^25^ showed that organic matter significantly improved soil porosity and water content in sandy loams, and subsequently increased soil carbon, nitrogen and phosphorus content. Therefore, planting fast-growing poplar and applying organic fertiliser can be used in the reclamation of abandoned homesteads.

Land restoration measures greatly influence the extent and direction of soil structure quality changes^26,27^. Soil contains irregular particles and voids^28^ and has a complex structure. Traditional soil texture analysis methods describe soil particle distribution (PSD) based on the percentage of different particle contents in the total weight of the soil, which usually results in incomplete soil PSD information^29^. As the basic unit of soil structure, soil particles have a certain self-similarity, which fully satisfies the applicable conditions of fractal theory. Fractal theory is a method developed by Mandelbrot to effectively describe the local morphology, structure and information of complex geometric shapes in nature. Although some controversial views need to be further determined^30–33^, most of these studies^34–37^ have confirmed that fractal geometry is an effective tool for characterizing the features of soil PSD. Since Scott et al.^38^ and Yang Peiling et al.^39^ successfully applied fractal theory to describe the characteristics of soil particle size distribution, coupled with the popularity of laser particle size analyzer, the fractal theory system has since been gradually developed and improved. In particular, single fractal and multifractal theory have been applied to study the soil PSD. Recently, multifractal geometry has been increasingly applied to quantify the features of soil PSD, with the multifractal parameters being regarded as more valid for evaluating indices for PSD in many studies. Because the correlation between traditional texture classification results and soil physical and chemical properties is lower than that of fractal dimension parameters^40^, and fractal method is more suitable to describe the scale and variability of soil properties, such as general soil properties: Soil nutrients, physical properties, water retention properties and pore size distribution characteristics^34,41^. Therefore, the fractal dimension parameter of soil is more suitable as an index to reflect soil degradation or restoration^42^. However, to date, it is rare to use fractal methods to assess the effectiveness of soil restoration.

Here, this study took abandoned homestead as a research object, reclaimed and planted fast-growing poplar through field tests, and explored the reclamation of abandoned homestead into poplar forest in Weicheng District, Xianyang City, Shaanxi Province, based on the fractal theory of soil particle size. Moreover, the soil particle size distribution was different under different forest management measures (chemical fertilizer + irrigation, chemical fertilizer + organic fertilizer + irrigation, irrigation and contrast, i.e. no planting of fast-growing poplar abandoned treatment). In addition, we also clarified the relationship between the basic physical and chemical properties of the soil and soil PSD under the reclaimed poplar forest and different forest management measures. The purpose of this study is to seek reasonable reclamation mode and land restoration measures for abandoned homesteads, as well as to explore the feasibility of evaluating the reclamation quality of abandoned homesteads from the perspective of multiple fractals of soil particle size distribution, so as to provide a scientific basis for alleviating the contradiction between land supply and demand and to ensure the long-term development of regional ecology. The specific research objectives were as follows:

- Basic characteristics of soil particle size under poplar forest reclamation and different management measures
- The multi-fractal characteristics of soil particle size under poplar forest reclamation and different management measures
- Correlation between soil physical and chemical properties and soil multifractal parameters under poplar forest reclamation land and different management measures.

## Materials and methods

### Overview of the study area

The study area is located in Xixian New District of Shaanxi Province (E 107° 38′ –109° 10′, N 34° 9′–35° 34′). It exhibits a varying topography, with higher elevations in the north and lower elevations in the south. The northern part is the southern edge of the semi-arid gully region of the Weibei Loess Plateau. In contrast, the southern part belongs to the Guanzhong Plain and is characterized by the Weihe Basin. This region has a warm temperate climate with distinct four seasons. The mild climate, abundant light, heat, and water resources create favorable conditions for agriculture, forestry, animal husbandry, and fishing industries. The average annual precipitation ranges from 537 to 650 mm, while the average temperature ranges from 9.0 to 13.2 °C. In addition, the area enjoys a high average cumulative annual sunshine duration of 2017.2 to 2346.9 h. In particular, the months of June, July, and August contribute to about 32 of total annual sunshine hours, which promotes the ripening of summer crops and the growth and development of autumn crops. Furthermore, the frost-free period ranges from 172 to 205 days in the north and from 212 to 223 days in the south^43^.

### Reclamation model of abandoned homestead

The experimental area selected for this study was previously an abandoned homestead. This area underwent village withdrawal, expansion, and consolidation, after which the reclamation process took place. Reclamation involved several steps, including bulldozing the area, clearing the surface, and ploughing. To start the reclamation process, fast-growing poplar trees (European and American 107) were selected for planting. In April 2018, one-year-old poplar seedlings were carefully selected based on their diameter at breast height of 2.8 m, seedling height of 3.0 m, healthy growth, and a complete root system. The planting layout consisted of trees spaced 1.0 m apart in rows, with 0.5 m between each plant. The total area planted was 3.07 hm^2^.

In May 2018, plots with essentially the same poplar growth were selected to delimit the experimental plots. The experimental design included four treatments: (1) irrigation only (Irrigation); (2) chemical fertilizer application + irrigation (Fertilizer irrigation); (3) chemical fertilizer application + organic fertilizer + irrigation (Mixed fertilizer irrigation); and (4) a control group representing abandoned land that had been bulldozed, cleared and ploughed but did not receive any poplar planting, fertilization, or irrigation (Abandoned land).Each treatment was replicated three times, and the plot size was 60 m^2^ (15 m × 4 m). The irrigation method was flood irrigation, and the irrigation volume of a single experimental plot was 4 m^3^. Fertilization was carried out with 0.25 kg of urea and 0.5 kg of superphosphate per plant. In addition, organic fertilizer was applied in the form of 8 kg of manure per plant. Fertilization was carried out by digging four evenly spaced holes around each poplar tree to a depth of approximately 20 cm. During the later stages, fertilizer irrigation was mainly and mixed fertilizer irrigation was treated with top dressing, with 0.5 kg urea per plant for fertilizer irrigation and 0.5 kg urea + 2 kg manure per plant for mixed fertilizer irrigation. Irrigation was applied immediately after fertilization, and the frequency of fertilization and irrigation was once in May and July each year.

### Soil sampling

**Figure 1:**
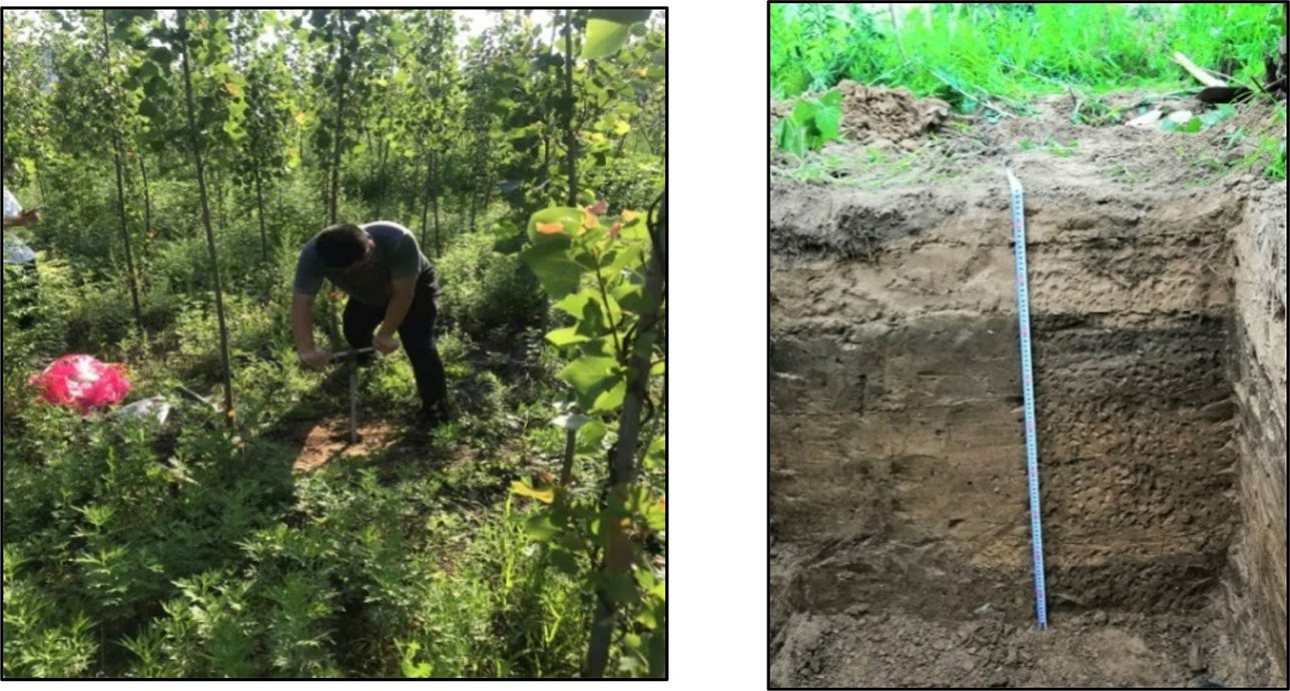
Soil sample collection and Soil profile.

In September 2020, soil sampling was carried out using a diagonal method, with five points selected within each test plot. A 40 mm diameter soil drill was used for sampling (Fig. 1), with the depths of 0–20 cm, 20–40 cm, 40–60 cm, 60–80 cm and 80–100 cm. After removing plant roots and gravel, some of the soil samples were collected and placed in an aluminium box to determine soil moisture content, and some were placed in a self-sealed bag and brought back to the experiment for later use. The soil samples brought back to the laboratory were air-dried for 7 days and then passed through a 2 and 0.25 mm sieve, respectively. In addition, a1m profile measuring 1m in depth was excavated from each treatment to investigate soil properties across different genetic horizons and to collect soil bulk density samples (Fig. 1).

### Laboratory measurements

The laser particle analyzer (Mastersizer 2000, Malvern Company, UK) was used to measure the percent volume of soil particles in the range 0.02–2000 μm. According to the US classification standards, soil particles are divided into three classes: clay particles < 0.002 mm, silt particles 0.002–0.05 mm and sand 0.05–2.000 mm.

Soil bulk density (BD) and soil moisture contents (SM) were measured using a gravimetric method^43^. The conventional experimental method was used to determine soil organic matter (SOM), soil total nitrogen (STN), soil available phosphorus (SAP) and soil available potassium (SQP)^44^. The basic physical and chemical properties of the soil in the study area are shown in Table 1.

**Table 1:** Physiochemical properties of soil (mean ± one standard deviation) in the 0–100 cm layer under different management measures.

Management measures	Soil depth (cm)	SOM (g kg^−1^)	STN (g kg^−1^)	SAP (mg kg^−1^)	SQP (mg kg^−1^)	BD (g cm^−3^)	SM (%)	pH
Abandoned land	0–20	7.50 ± 1.00	0.41 ± 0.04	8.29 ± 0.11	109 ± 15	1.56 ± 0.05	8.20 ± 0.43	8.82 ± 0.22
	20–40	7.15 ± 1.17	0.43 ± 0.03	7.10 ± 0.48	113 ± 16	1.64 ± 0.04	7.77 ± 0.51	8.79 ± 0.55
	40–60	7.08 ± 1.66	0.44 ± 0.06	6.88 ± 1.75	127 ± 20	1.57 ± 0.04	7.55 ± 0.36	8.88 ± 0.52
	60–80	6.36 ± 0.38	0.42 ± 0.04	8.28 ± 1.29	124 ± 3	1.49 ± 0.06	7.95 ± 0.65	8.74 ± 0.66
	80–100	5.16 ± 0.33	0.34 ± 0.06	7.30 ± 1.68	114 ± 16	1.45 ± 0.01	8.36 ± 0.35	8.96 ± 0.50
Irrigation	0–20	10.09 ± 2.40	0.59 ± 0.03	9.20 ± 3.49	230 ± 66	1.51 ± 0.02	10.18 ± 0.51	8.77 ± 0.37
	20–40	9.09 ± 6.26	0.65 ± 0.06	8.44 ± 0.99	137 ± 48	1.61 ± 0.03	12.92 ± 1.02	8.73 ± 0.38
	40–60	8.41 ± 2.03	0.56 ± 0.08	13.77 ± 7.64	178 ± 67	1.44 ± 0.02	11.95 ± 1.32	8.72 ± 0.01
	60–80	6.36 ± 1.20	0.53 ± 0.04	8.16 ± 1.50	157 ± 22	1.58 ± 0.06	11.33 ± 0.87	8.73 ± 0.02
	80–100	7.11 ± 0.78	0.53 ± 0.03	8.50 ± 1.08	191 ± 48	1.64 ± 0.05	12.27 ± 0.54	8.64 ± 0.10
Fertilizer irrigation	0–20	14.63 ± 1.73	1.24 ± 0.18	20.6 ± 3.81	287 ± 12	1.44 ± 0.04	13.04 ± 1.33	8.65 ± 0.09
	20–40	10.12 ± 0.42	0.70 ± 0.02	8.1 ± 0.77	138 ± 7	1.63 ± 0.06	13.59 ± 1.24	8.68 ± 0.03
	40–60	9.95 ± 0.40	0.62 ± 0.04	6.84 ± 0.30	149 ± 25	1.59 ± 0.05	12.50 ± 0.98	8.64 ± 0.08
	60–80	7.99 ± 0.37	0.61 ± 0.02	9.70 ± 2.49	162 ± 10	1.52 ± 0.04	12.14 ± 0.77	8.85 ± 0.03
	80–100	7.50 ± 1.07	0.58 ± 0.04	10.40 ± 2.11	199 ± 6	1.45 ± 0.02	12.39 ± 0.74	8.85 ± 0.30
Mixed fertilizer irrigation	0–20	18.36 ± 4.90	0.63 ± 0.03	11.97 ± 5.12	347 ± 32	1.41 ± 0.01	13.11 ± 0.88	8.58 ± 0.31
	20–40	7.89 ± 0.03	0.55 ± 0.03	9.94 ± 1.91	121 ± 15	1.56 ± 0.07	13.10 ± 1.12	8.52 ± 0.08
	40–60	6.54 ± 0.29	0.49 ± 0.03	7.63 ± 1.04	104 ± 11	1.55 ± 0.06	11.71 ± 1.31	8.51 ± 0.16
	60–80	8.03 ± 0.02	0.57 ± 0.01	6.74 ± 1.94	127 ± 7	1.55 ± 0.03	11.85 ± 0.96	8.71 ± 0.19
	80–100	8.20 ± 0.79	0.56 ± 0.03	3.99 ± 0.53	138 ± 1	1.36 ± 0.02	12.59 ± 0.54	8.74 ± 0.39

SOM: Soil organic matter; STN: Soil total nitrogen; SAP: Soil available phosphorus; SQP: Soil available potassium; BD: Soil bulk density; SM: Soil moisture; pH: Soil pH.

### Fractal principle of soil particle size

#### Calculation of the single fractal dimension

The soil particle volume fractal model was used^38,39^ and the single fractal dimension (*D*) of the soil PSD was calculated using the formula (1):

1 $$\frac{{V_{(r < R)} }}{{V_{T} }} = \left( {\frac{R}{{ \lambda_{v} }}} \right)^{3 - D}$$

where $V_{(r < R)}$ is the cumulative volume of soil particles with a particle size smaller than R; $V_{T}$ represents the total volume of the soil particles; *R* is a specific particle size feature scale, μm , the arithmetic mean of the upper and lower limits of a particle size interval is taken for calculation; $\lambda_{v}$ is the maximum particle size in the soil particle size classification, μm; *D* represents the single fractal dimension of the soil particle size distribution dimensionless. The *D* from small to large can represent the change in soil texture from coarse to fine or from loose to dense and can be used to describe the overall roughness of the soil particles. The smaller the *D* value, the rougher the soil particles as a whole.

#### Multifractal theory

According to the measuring range *I* = [0.2 2000] μm of the laser particle size analyzer, which is divided into 2^k^ sub-intervals in a logarithmically increasing and equivalent manner. That is, the endpoint values of each subinterval *I*_*i*_ = (ψ_*i*_, ψ_*i*+*1*_) and lg (ψ_*i*+*1*_ /ψ_*i*_) = ε, where ε is a constant. The first sub-interval was *I*_*1*_ = [0.02, 0.024], and the 64th sub-interval was *I*_*64*_ = [1670.725, 2000]. To ensure that each sub-interval contains at least one measurement value after division,* k* is set to1 ~ 6 in this study. Construct the dimensionless interval *J* = [0, lg (2000/0.02)] = [0, 5], and divide the interval J into 2^k^ sub-interval *J*_*i*_ with the same interval epsilon. If the number of intervals is N = 2^k^, ε = 5 × 2^−k^. *v*_*i*_ is the soil volume fraction in subinterval *I*_*i*_, and *μ*_i_ (ε) is the probability density of the soil particle size distribution in the subinterval *J*_*i*_^36,38,39^.

Construct a family of partition functions:

2 $$\mu_{i} \left( {q,\varepsilon } \right) = \frac{{\mu_{i} \left( \varepsilon \right)^{q} }}{{\mathop \sum \nolimits_{i = 1}^{N\left( \varepsilon \right)} \mu_{i} \varepsilon^{q} }}$$

where $\mu_{i} \left( {q,\varepsilon } \right)$ is the Q-order probability on the ith interval, the step size q is an integral real number and its value range is [− 10, 10], then the generalized fractal dimension (D(q)) of the multifractal of the soil particle size distribution is:

3 $$D\left( q \right) = \frac{1}{q - 1}\mathop {{\text{lim}}}\limits_{\varepsilon \to 0} \frac{{\mathop {\mathop \sum \limits^{N\left( \varepsilon \right)} }\limits_{i = 1} \mu_{i} \left( \varepsilon \right)^{q} }}{\lg (\varepsilon )} \left( {q \ne 1} \right)$$

4 $$D\left( 1 \right) = \mathop {{\text{lim}}}\limits_{\varepsilon \to 0} \frac{{\mathop {\mathop \sum \limits^{N\left( \varepsilon \right)} }\limits_{i = 1} \mu_{i} \left( \varepsilon \right)\lg \mu_{i} \varepsilon }}{\lg (\varepsilon )} \left( {q = 1} \right)$$

According to formulae (2) and (3), when *q* = 0, *D*_*0*_ is the capacity dimension, *D*_*0*_ reflects the width range of the soil particle size distribution. The larger the D_0_ value is, the larger the width range of the soil particle size distribution is. When *q* = 1, *D*_*1*_ is the entropy dimension of the measurement, *D*_*1*_ value is between 0 and 1, the larger the value of *D*_*1*_, the wider the particle size range measured in the local distribution, the higher the degree of dispersion, and the more uniform the soil particle size distribution. When *q* = 2, *D*_*2*_ is the correlation dimension that describes the uniformity of soil particle size across measurement intervals, with larger values indicating a more uniform distribution of soil particle size across measurement intervals. When *q* <  − 1, information with low concentration or low aggregation is amplified; when *q* > 1, information with high concentration or high aggregation is amplified. *D*_*1*_*/D*_*0*_ is used to reflect the degree of dispersion of the soil particle size distribution. The closer its value is to 1, the more soil particles are distributed in dense areas, and the closer it is to 0, the more soil particles are distributed in sparse areas. When *q* < 0, *D*_*−10*_*–D*_*0*_ describes the fractal structure characteristics of soil, and the smaller the numerical value, the simpler the soil fractal structure; when *q* > 0, *D*_*0*_*–D*_*10*_ describes the overall inhomogeneity of soil particle size distribution, and the larger the value, the higher the overall inhomogeneity of soil particle size distribution^45,46^.

The formula for calculating the multifractal singularity index of soil particle size distribution is:

5 $${ }\alpha {\text{q}} = \mathop {\lim }\limits_{\varepsilon \to 0} \frac{{\mathop {\mathop \sum \limits^{N\left( \varepsilon \right)} }\limits_{i = 1} \mu_{i} \left( {q,\varepsilon } \right)\lg \mu_{i} \left( \varepsilon \right)}}{lg\varepsilon }$$

The formula for calculating the multifractal singularity spectra of the soil particle size distribution is:

6 $${ }f\left( {\alpha {\text{q}}} \right) = \mathop {\lim }\limits_{\varepsilon \to 0} \frac{{\mathop {\mathop \sum \limits^{N\left( \varepsilon \right)} }\limits_{i = 1} \mu_{i} \left( {q,\varepsilon } \right)lg\mu_{i} \left( {q,\varepsilon } \right)}}{lg\varepsilon }$$

The parameters of *α(q)* and *f(α)* can characterize the local multifractal characteristics of the soil particle size distribution. *α(0)* is the mean value of the multifractal singular spectrum. The greater the *α(0)* means the lower the local density of the soil particle size distribution. *Δα(q)* represents the spectral width of the multifractal spectrum, which reflects the degree of heterogeneity and spatial heterogeneity of soil properties. The larger the *Δα*, the more uneven the distribution, which is defined as follows:

7 $$\Delta \alpha \left( q \right) = \alpha \left( q \right)_{{{\text{max}}}} - \alpha \left( q \right)_{{{\text{min}}}}$$

where the min and max are the minimum and maximum values, respectively. The shape of the multifractal singular spectrum *Δf* [α(q)] is defined as follows:

8 $$\Delta f \left[ {\alpha \left( q \right)} \right] = f \left[ {\alpha \left( q \right)_{max} } \right] - f \left[ {\alpha \left( q \right)_{min} } \right]$$

*Δf(α)* characterizes the shape characteristics of the multifractal spectrum, and *Δf(α)* < 0 indicates that small particle size plays an important role in the soil particle size distribution, and the variability of small particle size is greater than that of large particle size. *Δf(α)* > 0 indicates that large particle size plays an important role in soil particle size distribution, and the variability of large particle size is greater than that of small particle size^47,48^.

### Statistical analysis

Malvern 3000 software was used for granular data export; Microsoft Excel 2010 software was used for basic data statistics and processing; the software IBM Statistics SPSS 22 software was used for one-way analysis of variance and LSD method was for significance test. Pearson correlation analysis and stepwise regression analysis. The software Origin 2018 software was used to draw the soil texture triangle map and multifractal map.

### Ethical approval

No animal studies are presented in this manuscript. No human studies are presented in this manuscript. No potentially identifiable human images or data is presented in this study.

## Results

### Basic characteristics of soil particle size distribution

**Figure 2:**
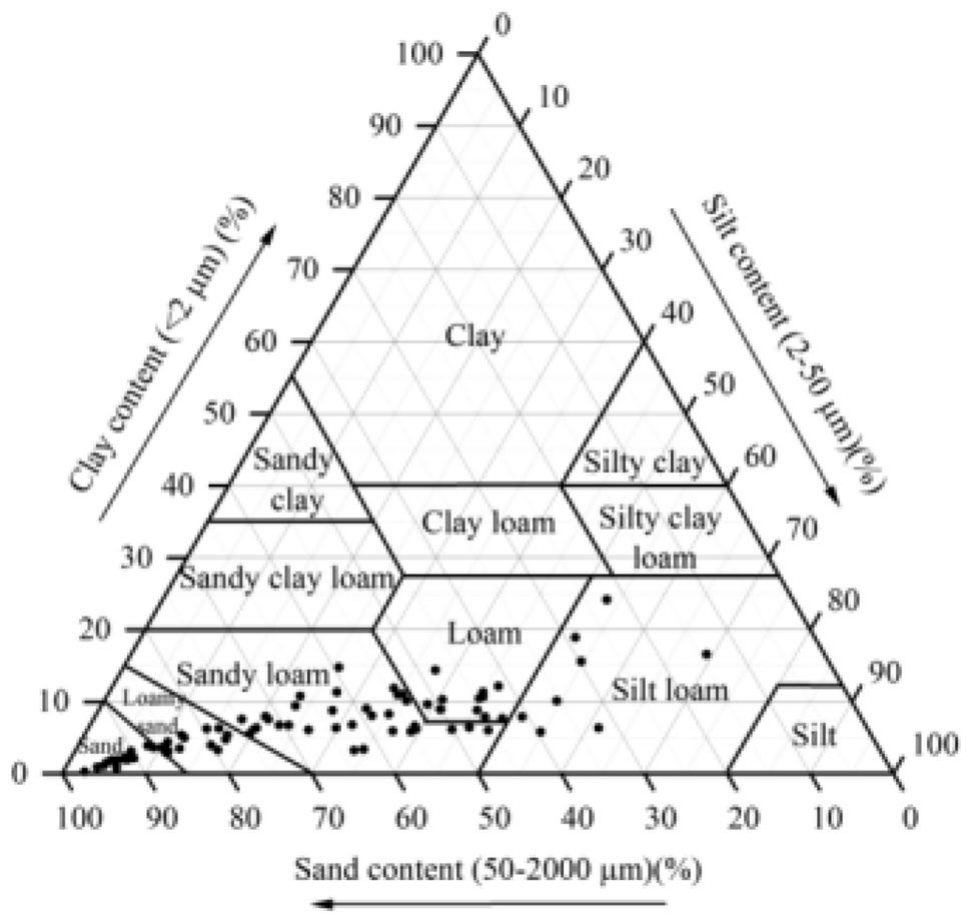
Texture of analyzed soil samples.

The soil texture distribution (Fig. 2) reveals that soil the texture types in the study area are mainly silt loam and silty soils. According to Table 2, the clay content starts from the 40 cm soil layer and increases with increasing soil depth under irrigation, fertilizer irrigation and mixed fertilizer irrigation. The clay content ranges from 13.70 to 15.88% with an average of 14.58. The silt content ranges from 80.20 to 82.28% with an average of 82.21%. The sand content ranges from 1.85 to 6.11% with an average of 4.22%. Compared with the abandoned land, the clay and silt content under irrigation mode decreased by 3.40 and 1.59%, respectively, while the average sand content increased by 42.04%. Under fertilizer irrigation and mixed fertilizer irrigation modes, the clay content increased by 4.01 and 14.89%, respectively, while the average silt and sand content decreased by 0.02%, 13.70%, and 1.24%, 27.82%, respectively. There was no significant difference in clay, silt and sand content between the different management treatments in the same soil layer. Under the fertilizer irrigation and mixed fertilizer irrigation modes, the average soil clay and silt contents showed significant differences among different soil layers (*P* < 0.05); under irrigation and abandonment modes, there was no significant difference in soil clay, silt, and sand contents among different soil layers.

**Table 2:** Soil particle composition and single fractal dimension under different management measures.

Management measures	Soil depth(cm)	Clay content (%)	Silt content (%)	Sand content (%)	D
Abandoned land	0 − 20	14.29 ± 1.20Ab	81.15 ± 2.50Aa	4.56 ± 0.88Aa	2.753 ± 0.01Bb
	20 − 40	14.58 ± 1.70Ab	82.17 ± 0.60Aa	3.26 ± 1.10Aa	2.755 ± 0.01Bb
	40 − 60	15.88 ± 1.77Aa	82.28 ± 1.28Aa	1.85 ± 0.49Aa	2.767 ± 0.01Ba
	60 − 80	14.46 ± 0.90Ab	80.24 ± 1.29Aa	5.31 ± 0.77Aa	2.754 ± 0.01Bb
	80 − 100	13.70 ± 2.43Bc	80.20 ± 5.00Aa	6.11 ± 0.32Aa	2.747 ± 0.02Bb
	Mean value	14.58 ± 0.8Ba	81.20 ± Aa	4.22 ± 1.69Ba	2.755 ± 0.01Bb
Irrigation	0 − 20	12.01 ± 1.98Aa	78.82 ± 3.79Aa	9.18 ± 0.46Aa	2.713 ± 0.07Bc
	20 − 40	10.23 ± 4.42Aa	78.74 ± 7.09Aa	11.04 ± 0.19Aa	2.721 ± 0.03Bc
	40 − 60	13.37 ± 0.86Aa	81.74 ± 1.51Aa	4.90 ± 0.95Aa	2.744 ± 0.01Bb
	60 − 80	16.27 ± 5.30Aa	80.29 ± 0.74Aa	3.45 ± 0.30Aa	2.775 ± 0.03Ba
	80 − 100	18.56 ± 0.29Aa	80.03 ± 1.03Aa	1.42 ± 0.10Aa	2.787 ± 0Ba
	Mean value	14.09 ± 3.33Ba	79.92 ± 1.23Ba	6.00 ± 3.79Aa	2.748 ± 0.05Bb
Fertilizer irrigation	0 − 20	10.77 ± 0.97Ab	81.74 ± 6.39Aa	7.5 ± 0.28Aa	2.726 ± 0Ac
	20 − 40	12.32 ± 2.41Ab	81.72 ± 1.30Aa	5.97 ± 0.53Aa	2.755 ± 0.01Ab
	40 − 60	14.85 ± 4.33Aab	82.88 ± 2.49Aa	2.28 ± 0.43Aa	2.758 ± 0.01Ab
	60 − 80	18.61 ± 1.55Aa	80.26 ± 0.51Aa	1.14 ± 0.37Aa	2.808 ± 0.01Aa
	80 − 100	19.30 ± 0.53Aa	79.37 ± 1.29Aa	1.34 ± 0.40Aa	2.799 ± 0Aa
	Mean value	15.17 ± 3.76Ba	81.19 ± 1.38Aa	3.65 ± 2.76Ba	2.769 ± 0.03Ab
Mixed fertilizer irrigation	0 − 20	11.27 ± 0.54Ac	80.50 ± 0.01Aabc	8.23 ± 0.52Aa	2.721 ± 0.01Ac
	20 − 40	14.49 ± 1.44Ac	84.07 ± 0.33Aa	1.45 ± 0.37Ab	2.748 ± 0Ab
	40 − 60	15.97 ± 2.23Abc	82.36 ± 2.81Aab	1.68 ± 0.59Ab	2.781 ± 0Aa
	60 − 80	22.06 ± 3.16Aa	76.12 ± 3.45Ac	1.83 ± 0.29Ab	2.787 ± 0.01Aa
	80 − 100	19.98 ± 1.31Aab	77.97 ± 0.57Abc	2.06 ± 0.46Ab	2.789 ± 0Aa
	Mean value	16.75 ± 4.31Ab	80.20 ± 3.22Ba	3.05 ± 2.76Bb	2.765 ± 0.03Ab

Different uppercase letters represent the same soil layer, and there are significant differences among different management measures (*P* < 0.05); Different lowercase letters indicate the same management measures, and there are significant differences among different soil layers (*P* < 0.05).

At depths of soil 0–20 and 20–40 cm, *D* values were in the order abandoned land > fertilizer irrigation > mixed fertilizer irrigation > irrigation. At depths of soil 60–80 cm and 80–100 cm, *D* values were in the order of fertilizer irrigation > mixed fertilizer irrigation > irrigation > abandoned land, and were significantly higher under fertilizer and mixed fertilizer irrigation than under irrigation and abandoned land (*P* < 0.05).The* D* under the four land reclamation modes ranged from 2.721 to 2.808, and the mean value was in the order of irrigation (2.748) < abandoned land (2.755) < mixed fertilizer irrigation (2.765) < fertilizer irrigation (2.769), indicating that the overall roughness of the soil gradually decreased and fine particles increased in this order. In addition, the *D* under fertilizer irrigation and mixed fertilizer irrigation was significantly higher than that of the abandoned land and irrigation (*P* < 0.05), indicating that the soil particles were finely granulated by fertilization. Under mixed fertilizer irrigation and irrigation, *D* increased with the increase of soil depth, and reached the maximum value in 0–100 cm soil layer. However, the *D* value increased first and then decreased with the increase of soil depth under fertilizer irrigation and abandoned land.

### Multifractal characteristics of soil particle size distribution under different management modes

**Figure 3:**
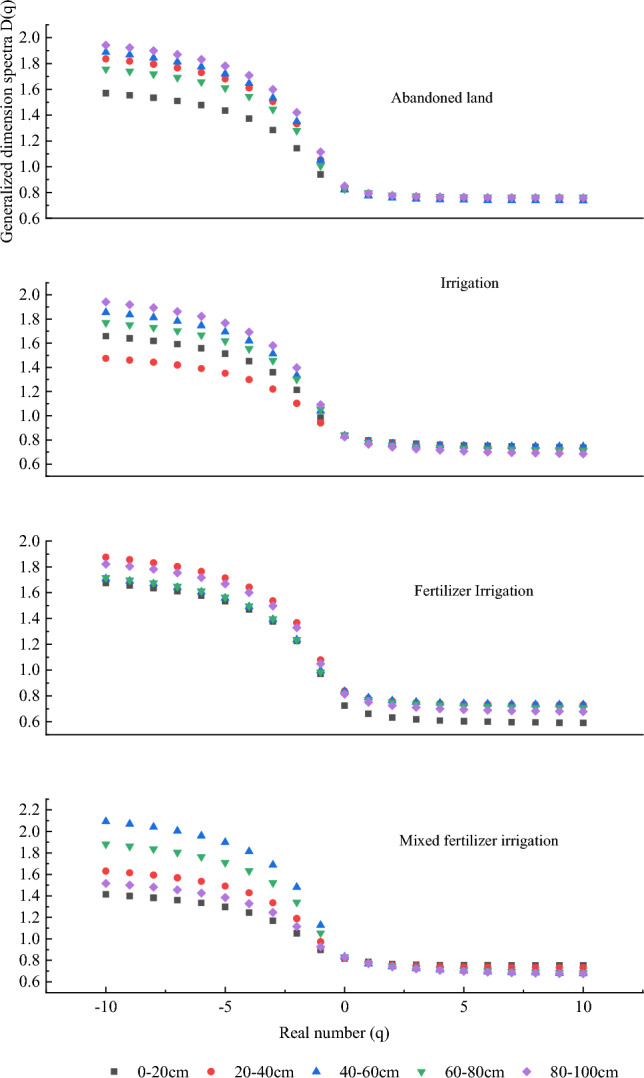
Distribution of generalised dimensional spectral curves *q* ~ *D(q)* of different soil layers under different management measures.

The parameters *D*_*0*_, *D*_*1*_, and *D*_*2*_ reflect the non-uniformity of the overall fractal structure of the soil. Generally, *D*_*0*_ ≥ *D*_*1*_ ≥ *D*_*2*_, and when *D*_*0*_ = *D*_*1*_ = *D*_*2*,_ it indicates that the soil particle size is uniformly distributed. The *q-D (q)* curves in the multiple fractal dimension plot of soil particle size in the poplar forest under different management modes in the study area (Fig. 3) reflect the uniformity of the soil. The smaller the curvature, the more uniform the soil particle size distribution. The multi-fractal dimension parameters (Table 3) show that the mean values of *D*_*0*_, *D*_*1*_ and *D*_*2*_ in abandoned land are 0.831, 0.788 and 0.771, respectively. For the fertilizer irrigated soil, the mean values of *D*_*0*_, *D*_*1*_, and *D*_*2*_ are 0.806, 0.750, and 0.727, respectively. For the mixed fertilizer irrigated soil, the mean values of *D*_*0*_, *D*_*1*_, and *D*_*2*_ are 0.826, 0.775, and 0.751, respectively. For the irrigated soil, the mean values of *D*_*0*_, *D*_*1*_, and *D*_*2*_ are 0.833, 0.783, and 0.764, respectively. It can be observed that the multifractal dimension parameters of soils at different depths under different management modes follow the order of *D*_*0*_ ≥ *D*_*1*_ ≥ *D*_*2*_. In addition, all the *q-D (q)* curves exhibit a decreasing inverse "*S*" pattern and have a certain width (Fig. 2), indicating that the distribution of soil particle size in the study area is uneven, thus requiring multifractal analysis.

**Table 3:** Multifractal dimensional parameters of soil particle size distribution under different management measures.

Management	Soil depth (cm)	*D*_0_	*D*_1_	*D*_2_	*D*_-10_—*D*_0_	*D*_0_—*D*_10_	*D*_1_/*D*_0_	Δα	*Δf*
Abandoned land	0 − 20	0.823 ± 0.02Bb	0.789 ± 0.02Aa	0.774 ± 0.02Aa	0.747 ± 0.4Bc	0.066 ± 0.03Cb	0.959 ± 0.03Aa	1.065 ± 0.07	0.984 ± 0.01
	20 − 40	0.832 ± 0.01Ab	0.783 ± 0.01Ab	0.766 ± 0.01Bb	1.003 ± 0.24Ab	0.083 ± 0.02Ba	0.941 ± 0.01Ab	1.351 ± 0.06	0.954 ± 0.01
	40 − 60	0.824 ± 0.01Ab	0.774 ± 0Bb	0.758 ± 0Ab	1.064 ± 0.24Ba	0.087 ± 0.01Ba	0.94 ± 0.01Ab	1.408 ± 0.12	1.016 ± 0.03
	60 − 80	0.829 ± 0.01Bb	0.797 ± 0.01Aa	0.781 ± 0.01Aa	0.928 ± 0.29Bc	0.067 ± 0.02Cb	0.962 ± 0.02Aa	1.261 ± 0.07	0.930 ± 0.03
	80 − 100	0.85 ± 0.03Aa	0.794 ± 0.04Aa	0.777 ± 0.04Aa	1.093 ± 0.14Aa	0.091 ± 0.01Ca	0.934 ± 0.01Ab	1.479 ± 0.10	0.950 ± 0.02
	Mean value	0.831 ± 0.02Ab	0.788 ± 0.02Ab	0.771 ± 0.02Ab	0.967 ± 0.28Ab	0.079 ± 0.02Bb	0.947 ± 0.02Ab	1.313 ± 0.16	0.967 ± 0.03
Irrigation	0 − 20	0.838 ± 0.05Aa	0.796 ± 0.04Aa	0.779 ± 0.05Aa	0.82 ± 0.45Bc	0.101 ± 0.05Bc	0.951 ± 0.04Aa	1.214 ± 0.07	0.990 ± 0.02
	20 − 40	0.828 ± 0.01Bc	0.789 ± 0.02Aa	0.771 ± 0.02Aa	0.646 ± 0.19Cc	0.105 ± 0.03Ac	0.953 ± 0.01Aa	1.093 ± 0.01	0.947 ± 0.03
	40 − 60	0.834 ± 0.01Ab	0.787 ± 0.01Aa	0.768 ± 0.01Ab	1.021 ± 0.22Ba	0.089 ± 0.01Bc	0.943 ± 0.01Ab	1.377 ± 0.06	1.002 ± 0.06
	60 − 80	0.839 ± 0.04Aa	0.78 ± 0.04Bb	0.759 ± 0.04Bb	0.93 ± 0.42Bb	0.123 ± 0.02Bb	0.93 ± 0.02Cc	1.303 ± 0.07	0.938 ± 0.01
	80 − 100	0.825 ± 0.03Ac	0.765 ± 0.02Cc	0.742 ± 0.02Bc	1.115 ± 0.31Aa	0.14 ± 0.02Ba	0.928 ± 0.01Ac	1.502 ± 0.04	0.988 ± 0.07
	Mean value	0.833 ± 0.03Ab	0.783 ± 0.03Ab	0.764 ± 0.03Ab	0.906 ± 0.35Bb	0.111 ± 0.03Ab	0.941 ± 0.02Ab	1.298 ± 0.16	0.978 ± 0.03
Fertilizer irrigation	0 − 20	0.725 ± 0.09Cc	0.661 ± 0.16Bc	0.633 ± 0.17Bc	0.949 ± 0.61Ab	0.133 ± 0.11Aa	0.901 ± 0.11Bb	1.312 ± 0.06	0.796 ± 0.02
	20 − 40	0.838 ± 0.05Aa	0.782 ± 0.04Aa	0.76 ± 0.03Ca	1.037 ± 0.28Aa	0.108 ± 0.02Ab	0.933 ± 0.02Ab	1.399 ± 0.02	0.978 ± 0.04
	40 − 60	0.834 ± 0.05Aa	0.786 ± 0.04Aa	0.764 ± 0.03Aa	0.874 ± 0.24Cc	0.103 ± 0.03Bb	0.942 ± 0.01Aa	1.214 ± 0.01	0.993 ± 0.02
	60 − 80	0.817 ± 0.01Cb	0.772 ± 0.02Bb	0.751 ± 0.02Cb	0.899 ± 0.3Bc	0.111 ± 0.02Bb	0.944 ± 0.02Ba	1.253 ± 0.04	0.983 ± 0.05
	80 − 100	0.813 ± 0.01Cb	0.751 ± 0.01Cb	0.726 ± 0Cb	1.009 ± 0.08Bb	0.135 ± 0.01Ba	0.924 ± 0.01Ab	1.401 ± 0.05	0.908 ± 0.01
	Mean value	0.806 ± 0.06Bb	0.75 ± 0.08Bb	0.727 ± 0.09Bb	0.953 ± 0.33Ab	0.118 ± 0.05Ab	0.929 ± 0.05Bb	1.316 ± 0.08	0.932 ± 0.08
Mixed fertilizer irrigation	0 − 20	0.817 ± 0Bb	0.785 ± 0.02Aa	0.768 ± 0.02Aa	0.597 ± 0.19Cc	0.063 ± 0.01Cb	0.961 ± 0.02Aa	0.903 ± 0.02	0.918 ± 0.04
	20 − 40	0.821 ± 0.01Bb	0.776 ± 0.01Ab	0.757 ± 0.01Cb	0.81 ± 0.29Bc	0.089 ± 0Bb	0.945 ± 0.01Ab	1.132 ± 0.03	0.945 ± 0.01
	40 − 60	0.832 ± 0.01Aa	0.767 ± 0.01Bb	0.741 ± 0.01Bc	1.259 ± 0.1Aa	0.156 ± 0.01Aa	0.922 ± 0.01Bc	1.679 ± 0.01	1.068 ± 0.02
	60 − 80	0.831 ± 0.01Aa	0.772 ± 0Bb	0.746 ± 0Cc	1.051 ± 0.09Ab	0.148 ± 0.01Aa	0.928 ± 0Cc	1.443 ± 0.05	1.045 ± 0.04
	80 − 100	0.83 ± 0.02Ba	0.775 ± 0.01Bb	0.741 ± 0.01Bc	0.686 ± 0.29Bc	0.154 ± 0.02Aa	0.934 ± 0.02Ac	1.050 ± 0.07	0.949 ± 0.02
	Mean value	0.826 ± 0.01Ab	0.775 ± 0.01Ab	0.751 ± 0.01Ab	0.88 ± 0.32Cb	0.122 ± 0.04Ab	0.938 ± 0.02Ab	1.241 ± 0.31	0.985 ± 0.07

Different capital letters indicate the same soil layer and there are significant differences among different management measures (*P* < 0.05); different lower case letters indicate the same management measures and there are significant differences among different soil layers (*P* < 0.05).

As can be seen from Table 3, the multi-fractal dimension parameters of soil particle size under different management modes are as follows: *D*_*0*_: fertilizer irrigation (0.806) < mixed fertilizer irrigation (0.826) < abandoned land (0.831) < irrigation (0.833), indicating that the distribution range of soil particle size gradually increased in this order. The distribution range of soil particle size under fertilizer irrigation and mixed fertilizer irrigation was smaller than that of abandoned land, while the distribution range of irrigated soil particle size was larger than that of abandoned land. *D*_*1*_: fertilizer irrigation (0.750) < mixed fertilizer irrigation (0.775) < irrigation (0.783) < abandoned land (0.788), the larger the value, the more uniform the distribution of soil particle size at all scales. *D*_*2*_: fertilizer irrigation (0.727) < mixed fertilizer irrigation (0.751) < irrigation (0.764) < abandoned land (0.771), the distribution of soil particles in the measured interval is more uniform in this order. *D*_*−10*_*–D*_*0*_: Abandoned land (0.967) > fertilizer irrigation (0.953) > irrigation (0.906) > mixed fertilizer irrigation (0.880), indicating that the overall fractal structure of the soil becomes progressively simpler in this order. *D*_*0*_*–D*_*10*_: mixed fertilizer irrigation (0.122) > fertilizer irrigation (0.118) > irrigation (0.111) > abandoned land (0.079), the higher the *D*_*0*_*–D*_*10*_ is, the greater the overall non-uniformity. The overall uniformity of mixed fertilizer irrigation, fertilizer irrigation and irrigation is gradually increasing and is lower than that of abandoned land. *D*_*1*_*/D*_*0*_: Abandoned land (0.947) > irrigation (0.941) > mixed fertilizer irrigation (0.938) > fertilizer irrigation (0.931), indicating that soil particles were distributed mostly in areas with dense grain size under fertilizer irrigation, and mostly in areas with sparse grain size under abandoned land.

### Multifractal singularity spectrum of soil particle size distribution under different management modes

**Figure 4:**
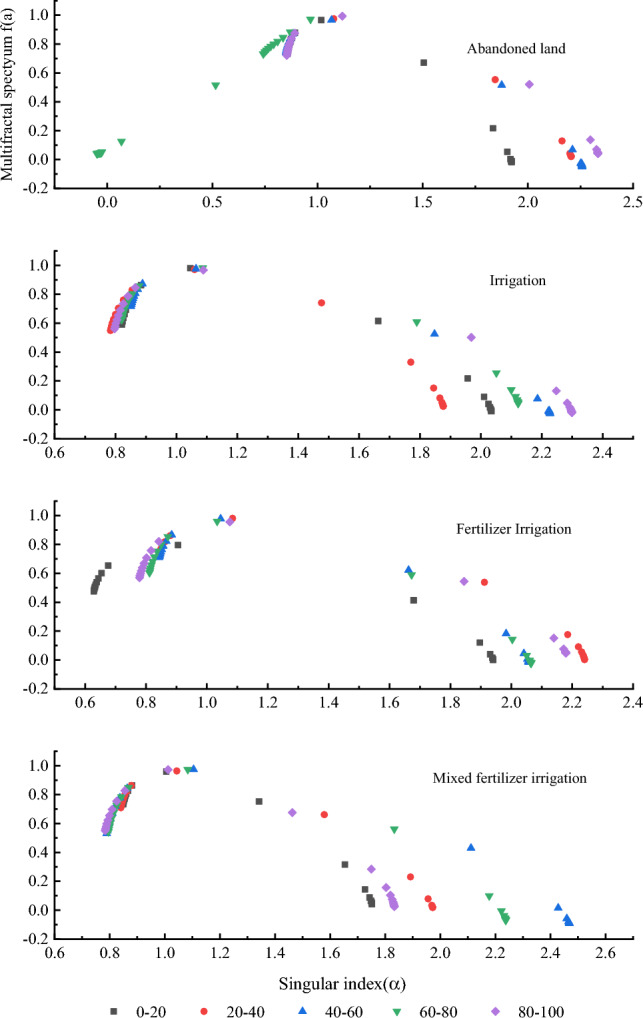
Multi-fractal Singular spectral function of soil particle size distribution under different management measures.

The multifractal dimension singularity curve can quantitatively reflect the spatial distribution information of soil particles, and the shape and symmetry of the curve reflect the heterogeneity of particle size distribution. Figure 4 shows that the soil singularity curves of different management modes are asymmetrical upward convex curves, indicating that the uneven distribution of soil particle size is caused by different degrees of superposition during soil formation.

The maximum value of *f (α(q))* in the singularity curve of multifractal dimension is equal to *D*_*0*_, and the spectral width *Δα* reflects the heterogeneity of the probability measure distribution of physical quantities across fractal structure. The larger *Δα* is, the more heterogeneous is the distribution of soil particle size. As shown in Table 3, the maximum and minimum values of *Δα* in abandoned land were 1.479 (80–100 cm) and 1.065 (0–20 cm), respectively, with an average value of 1.312. The maximum and minimum values of *Δα* in soil under fertilizer irrigation were 1.400 (80–100 cm) and 1.214 (40–60 cm), respectively, with a mean value of 1.316. Under mixed fertilizer irrigation, the maximum and minimum values of soil *Δα* were 1.679 (40–60 cm) and 0.903 (0–20 cm), respectively, with a mean value of 1.241. Under irrigation, the maximum and minimum values of soil *Δα* were 1.502 (80–100 cm) and 1.065 (0–20 cm), respectively, with a mean value of 1.298. The results showed that the uneven degree of soil particle size distribution under different management methods was in the order of fertilizer irrigation > abandoned land > irrigation > mixed fertilizer irrigation. Besides mixed fertilizer irrigation, the uneven degree of soil at 80–100 cm depth was the highest.

Based on the analysis of Table 3 and Fig. 4 analysis, it is evident that the *Δf* values of the soil samples from the four reclamation modes exhibit a positive left hook curve. This suggests that the dominant subset of soil particle size distribution corresponds to a larger probability, with a more uniform distribution of soil particle size in dense areas compared to sparse areas. Furthermore, Fig. 4 clearly demonstrates that the left and right diameters show a greater disparity under irrigation, fertilizer irrigation, and mixed fertilizer irrigation modes as compared to abandoned land. This observation indicates that the soil particle size distribution is more uneven under these three modes.

### The relationship between fractal dimension and soil particle composition and physicochemical properties

Table 4 presents the results of the correlation analysis between the fractal dimension parameters and soil particle composition and physicochemical properties. The analysis reveals that *D* showed a highly significant positive correlation with clay and a highly significant negative correlation with sand. In addition, *D* showed a positive correlation with *D*_*0*_*–D*_*10*_ and a negative correlation with *SOM* and *SAP*. Furthermore, *D*_*0*_ showed a highly significant positive correlation with *D*_*1*_, *D*_*2*_, and *D*_*1*_/*D*_*0*_. *D*_*1*_ showed a highly significant positive correlation with *D*_*2*_ and *D*_*1*_/*D*_*0*_, but displayed a significant negative correlation with *D*_*−10*_*–D*_*0*_. Further analysis revealed that *D*_*2*_ was highly significantly positively correlated with *D*_*1*_, *D*_*1*_/*D*_*0*_, and *D*_*−10*_–*D*_*0*_, but significantly negatively correlated with *D*_*0*_–*D*_*10*_. Moreover, *D*_*−10*_–*D*_*0*_ displayed a highly significant positive correlation with *Δα*, a highly negative correlation with *D*_*1*_/*D*_*0*_ and *SAP*, and a negative correlation with sand. Additionally, *D*_*0*_–*D*_*10*_ exhibited a highly significant positive correlation with clay, a highly negative correlation with *D*_*1*_/*D*_*0*_, and a negative correlation with *Δα* and *SM*. Finally, Δα displayed a highly significant negative correlation with *D*_*1*_/*D*_*0*_, *STN*, and *SAP*.

**Table 4:** Relationship between the fractal dimension of soil particle size distribution and soil particle composition and soil physicochemical properties.

	D	D_0_	D_1_	D_2_	D_−10_–D_0_	D_0_–D_10_	D_1_/D_0_	Δα	Δf
D	1								
D_0_	0.174	1							
D_1_	− 0.025	0.936**	1						
D_2_	− 0.096	0.901**	0.991**	1					
D_–10_–D_0_	0.429	0.081	− 0.176	− 0.181	1				
D_0_–D_10_	0.554*	− 0.172	− 0.447*	− 0.542*	0.377	1			
D_1_/_D0_	− 0.337	0.500*	0.773**	0.812**	− 0.580**	− 0.774**	1		
Δα	0.416	0.097	− 0.177	− 0.189	0.984**	0.469*	− 0.611**	1	
Δf	0.343	0.707**	0.604**	0.567**	0.367	0.116	0.220	0.378	1
Clay	0.908**	0.215	0.031	− 0.050	0.321	0.571**	− 0.277	0.316	0.386
Silt	− 0.194	− 0.145	− 0.104	− 0.048	0.187	− 0.384	0.001	0.085	− 0.063
Sand	− 0.866**	− 0.143	0.032	0.084	− 0.465*	− 0.380	0.301	− 0.396	− 0.380
SOM	− 0.550*	− 0.115	− 0.002	0.015	− 0.396	− 0.131	0.210	− 0.386	− 0.089
STN	− 0.278	− 0.143	− 0.052	− 0.061	− 0.439	− 0.049	0.149	− 0.452*	− 0.141
SAP	− 0.453*	− 0.076	0.106	0.106	− 0.662**	− 0.298	0.392	− 0.673**	− 0.193
SQP	− 0.346	− 0.281	− 0.210	− 0.219	− 0.377	0.100	− 0.002	− 0.352	− 0.251
BD	0.210	− 0.046	− 0.077	− 0.057	0.176	− 0.058	− 0.096	0.161	0.024
SM	0.004	0.061	− 0.010	− 0.073	− 0.230	0.454*	− 0.118	− 0.150	0.095
pH	0.324	− 0.010	− 0.091	− 0.100	0.228	0.035	− 0.215	0.160	− 0.010

** Represents significant difference at the 0.01 level (*p* < 0.01). * Represents significant difference at the 0.05 level (*p* < 0.05).

Stepwise regression results (Table 5) showed that clay and silt could jointly explain 85% of the variation in D value, indicating that clay and silt are the main controlling factors of *D* value variation. *SAP* could independently explain 44.9% of the variation in *D*_*−10*_–*D*_*0*_ and serves as the main controlling factor for this parameter. Furthermore, the combination of clay and *SM* explains 40.7% of the variation in *D*_*0*_–*D*_*10*_ and represents the main controlling factors for this aspect. Additionally, rapidly-available potassium accounts for 42.3% of the variation in *Δα*, indicating its importance as the main controlling factor for this parameter.

**Table 5:** Linear regression models of fractal parameters and basic soil properties.

Regression model	R^2^	*P*
D = 2.361 + 0.008Clay + 0.003Silt	0.852	< 0.001
D_−10_–D_0_ = 1.227–0.033SQP	0.449	0.002
D_0_–D_10_ = − 0.035 + 0.005Clay + 0.603SM	0.407	0.001
Δα = 1.622–0.036SQP	0.423	0.001

*SQP*: Soil available potassium; *SM*: Soil moisture.

## Discussion

### Soil particle size distribution and single fractal dimension under different reclamation modes

The study showed a decreasing trend in the average soil clay content in the Irrigation mode and an increasing trend in the average soil clay content in the fertilizer irrigation and mixed fertilizer irrigation mode compared to the abandoned land. This is mainly due to watering causing fine particles to move downwards, reducing the surface clay particle content, which is consistent with previous research showing that artificial irrigation or rainfall dilutes organic matter concentrations, transports large numbers of small particles, reduces internal forces between soil particles and reduces soil erosion resistance^49^. While, the application of organic fertilizers and manures can promote the growth and development of above and below ground plant parts, increase soil organic matter content, improve soil structure and retain fine particles such as sticky and powdery grains in the soil^50,51^.

The variation in *D* was observed to be different in different soil layers under the four reclamation modes. In the 0–20 cm and 20–40 cm soil layers, abandoned land had the highest *D* value, while irrigation, fertilizer irrigation, and mixed fertilizer irrigation modes had lower *D* values. This pattern indicates a decrease in the presence of small granular material in the soil under these three reclamation modes, mainly due to the downward movement of fine particles in the surface soil caused by flood irrigation. Conversely, in the 60–80 cm and 80–100 cm soil layers, abandoned land had the lowest *D* value, while irrigation, fertilizer irrigation, and mixed fertilizer irrigation modes had higher *D* values. This suggests an increase in the presence of fine-grained material in the deep soil. The main reason for this observation is the increase in clay content in the deep soil resulting from the effects of fertilization and irrigation infiltration, consequently leading to an increase in *D* values. This trend is consistent with the observed increase in clay content with increasing soil depth below the 40 cm soil layer.

### Multiple fractal dimensions of soil under different reclamation modes

*D*_*0*_, *D*_*1*_ and *D*_*2*_ are used as indicators to quantify the characteristics of soil *PSD* and reflect the degree of inhomogeneity of the overall fractal dimension distribution. There are differences in the effects of different management types on the uniformity and fractal structure of the soil particle size distribution. Taking the abandoned land soil as a control, mixed fertilizer irrigation has the most uniform soil texture, followed by fertilizer irrigation, and the uniformity of irrigated soil texture is the worst, all of which are higher than the abandoned land soil. According to the order of fertilizer irrigation, irrigation and mixed fertilizer irrigation, the overall soil structure tends to be simple. This is mainly influenced by the leaching and carrying effects of the downward movement of irrigation water, the large amount of farmyard manure application, and the effect of particle mixing^52,53^. Relevant studies^54^ have shown that the decomposition of vegetation litter, the crushing, interpenetration and encircling effects of roots on the soil^42^, and the gaps left by root death and microbial activity can change the distribution structure of soil particles. Small soil particles migrate vertically downwards with water infiltration and are trapped and deposited in soil layers with dense vegetation roots. Under irrigation, fertilizer irrigation and mixed fertiliser irrigation, the development status of vegetation roots is sequentially improved and soil resistance is stronger, leading to a gradual increase in soil texture uniformity in this order.

The mean *Δα* of fertilizer irrigation, mixed fertilizer irrigation, irrigation and abandoned land is 1.316, 1.241, 1.297, and 1.312, respectively. Guan Xiaoyan et al.^55^ in their study showed that the mean *Δα* of silt soil is 1.37. The difference between the *Δα* value in this study and previous studies may be due to the fact that the soil in the study area contains not only silty loam, but also some silty soil, which leads to a higher soil uniformity than that of silty loam. Therefore, the soil Δα in this study is lower compared to the results of Guan Xiaoyan et al.

### Relationship between fractal dimension of soil particle size distribution and basic soil properties

The fractal dimension of soil is calculated based on detailed particle size data, which is related to soil texture^56^. In this study, the Fractal dimension of soil volume has different degrees of correlation with the content of soil clay and sand particles, which is basically consistent with previous studies^57,58^. Stepwise regression analysis showed (Table 5) that clay was the main controlling factor in the variation of *D* and *D*_*0*_–*D*_*10*_, indicating that with the increase of clay content, the soil particle distribution showed heterogeneity and irregularity. The multifractal dimension parameters *D*_*0*_, *D*_*1*_, *D*_*2*_, *D*_*1*_/*D*_*0*_ and *Δf* had no significant relationship with soil physicochemical properties, indicating that these parameters have greater spatial specificity in characterizing soil quality. However, *D*, *D*_*−10*_–*D*_*0*_ and *D*_*0*_–*D*_*10*_ were significantly correlated with soil physicochemical properties and soil particle composition. These parameters could be used as potential indicators to reflect soil reclamation quality, indicating that the multifractal dimension could provide a reference for measuring soil structure and quality.

## Conclusions

We investigated the fractal dimension of soil particle size distribution under four reclamation modes. The results showed that fertilizer irrigation and mixed fertilizer irrigation could improve soil clay content and reduce soil particle roughness. The multifractal dimension parameters indicate that compared to undisturbed soil, human management (fertilization and irrigation) results in soil particle size distribution in dense areas, more non-uniform soil particles, and simplified soil structure. Due to the short reclamation time of the experimental area, the poplar forest did not have an obvious effect on soil improvement, resulting in the relationship between the multifractal dimension parameters (*D*_*0*_, *D*_*1*_ and *D*_*2*_) and soil particle composition and physical and chemical properties is not significant. However, the fractal dimension parameters (*D*, *D*_*−10*_–*D*_*0*_, *D*_*0*_–*D*_*10*_) had a good relationship with soil particle composition and physicochemical properties and could be used as potential indicators to characterize soil reclamation quality. Therefore, the multifractal theory can be used to analyze the change characteristics of reclaimed soil structure. The results of this study provide a new opportunity to evaluate the impact of the reclamation quality of abandoned homestead from the perspective of *PSD*, and also provide a reference for other poor soil reclamation evaluation.

## Data Availability

The original contributions presented in the study are included in the article/Supplementary Material, and further inquiries can be directed to the corresponding authors.
